# Supplementary material for: GDF11 induces differentiation and apoptosis and inhibits migration of C17.2 neural stem cells via modulating MAPK signaling pathway
Source: PeerJ. 2018 Sep 4;6:e5524. doi: 10.7717/peerj.5524 (PMC6128255; doi:10.7717/peerj.5524)
Supplement: Table S1 [file peerj-06-5524-s001.docx]

Table S1. Primer sequence for qRT-PCR

| Primers | Primer sequences | | | cell markers | | | | |
| --- | --- | --- | --- | --- | --- | --- | --- | --- |
| GAPDH | F: GGCATTGCTCTCAATGACAA  R:TGTGAGGGAGATGCTCAGTG | | housekeeping gene | | | | | |
| Nestin | F:GGAGGGCAGAGAAGACAGTG  R: TGACATCCTGGACCTTGACA | | | | neural stem cell | | | |
| βⅢ-tubulin | F:GAATGACCTGGTGTCCGAGT R:CAGAGCCAAGTGGACTCACA | neuron | | | | | | |
| GFAP | F: CACGAACGAGTCCCTAGAGC  R: TCACATCACCACGTCCTTGT | | | | | | astroglia | |
| Cyclin D2 | F: CTGTGCGCTACCGACTTCAA  R:GCAGAGCTTCGATTTGCTCC | | | | | | | cell cycle |
| Cyclin D1 | F: GCGTACCCTGACACCAATC  R:ATGCTAGAGGTCTGCGAGGA | | | | | | | cell cycle |
| EGFR | F: GGGATTGGCCTATTCATGCG  R:AATGCTCCCGAACCCAGAAC | | | | | EGFR pathway | | |
| Smad2 | F: CTTGCCATTCACACCGCCAG  R: CGGCAATATATAACATGGGC | | | | TGF-β pathway | | | |
| Smad3 | F: AAGGCCATCACCACGCAGAACG  R: TCCCCAGCCTTTGACGAAGCTC | | | | TGF-β pathway | | | |

*All primers were synthesized by Beijing Genomics Institute (Shenzhen, China).
