# Supplementary material for: GDF11 induces differentiation and apoptosis and inhibits migration of C17.2 neural stem cells via modulating MAPK signaling pathway
Source: PeerJ. 2018 Sep 4;6:e5524. doi: 10.7717/peerj.5524 (PMC6128255; doi:10.7717/peerj.5524)
Supplement: Table S2 [file peerj-06-5524-s002.doc]

| Coordinate | Target/Control | Alternate Nomenclature | Phosphorylation Site Detected |
| --- | --- | --- | --- |
| A1, A2 | Reference Spots | — | — |
| A21,A22 | Reference Spots | — | — |
| B3,B4 | Akt1 | PKBα, RACα | S473 |
| B5,B6 | Akt2 | PKBβ, RACβ | S474 |
| B7,B8 | Akt3 | PKBγ, RACγ | S472 |
| B9,B10 | Akt pan | — | S473,S474,S472 |
| B11,B12 | CREB | — | S133 |
| B13,B14 | ERK1 | MAPK3, p44 MAPK | T202/Y204 |
| B15,B16 | ERK2 | MAPK1, p42 MAPK | T185/Y187 |
| B17,B18 | GSK-3α/β | GSK3A/GSK3B | S21/S9 |
| B19,B20 | GSK-3β | GSK3B | S9 |
| C3,C4 | HSP27 | HSPB1, SRP27 | S78/S82 |
| C5,C6 | JNK1 | MAPK8, SAPK1γ | T183/Y185 |
| C7,C8 | JNK2 | MAPK9, SAPK1α | T183/Y185 |
| C9,C10 | JNK3 | MAPK10, SAPK1β | T221/Y223 |
| C11,C12 | JNK pan | — | T183/Y185, T221/Y223 |
| C13,C14 | MKK3 | MEK3, MAP2K3 | S218/T222 |
| C15,C16 | MKK6 | MEK6, MAP2K6 | S207/T211 |
| C17,C18 | MSK2 | RSKβ, RPS6KA4 | S360 |
| D3,D4 | P38α | MAPK14, SAPK2A, CSBP1 | T180/Y182 |
| D5,D6 | P38β | MAPK11, SAPK2B, p38-2 | T180/Y182 |
| D7,D8 | P38δ | MAPK13, SAPK4 | T180/Y182 |
| D9,D10 | P38γ | MAPK12, SAPK3, ERK6 | T183/Y185 |
| D11,D12 | P53 | — | S46 |
| D13,D14 | p70 S6 Kinase | S6K1, p70α, RPS6KB1 | T421/S424 |
| D15,D16 | RSK1 | MAPKAPK1α, RPS6KA1 | S 380 |
| D17,D18 | RSK2 | ISPK-1, RPS6KA3 | S386 |
| D19,D20 | TOR | ___ | S2448 |
| E19,E20 | PBS | Control (-) | — |
| F1,F2 | Reference Spots | — | — |

Table S2. The details of the Human Phospho-MAPK Array coordinates.
