# Supplementary figures and images for: GDF11 induces differentiation and apoptosis and inhibits migration of C17.2 neural stem cells via modulating MAPK signaling pathway
Source: PeerJ. 2018 Sep 4;6:e5524. doi: 10.7717/peerj.5524 (PMC6128255; doi:10.7717/peerj.5524)

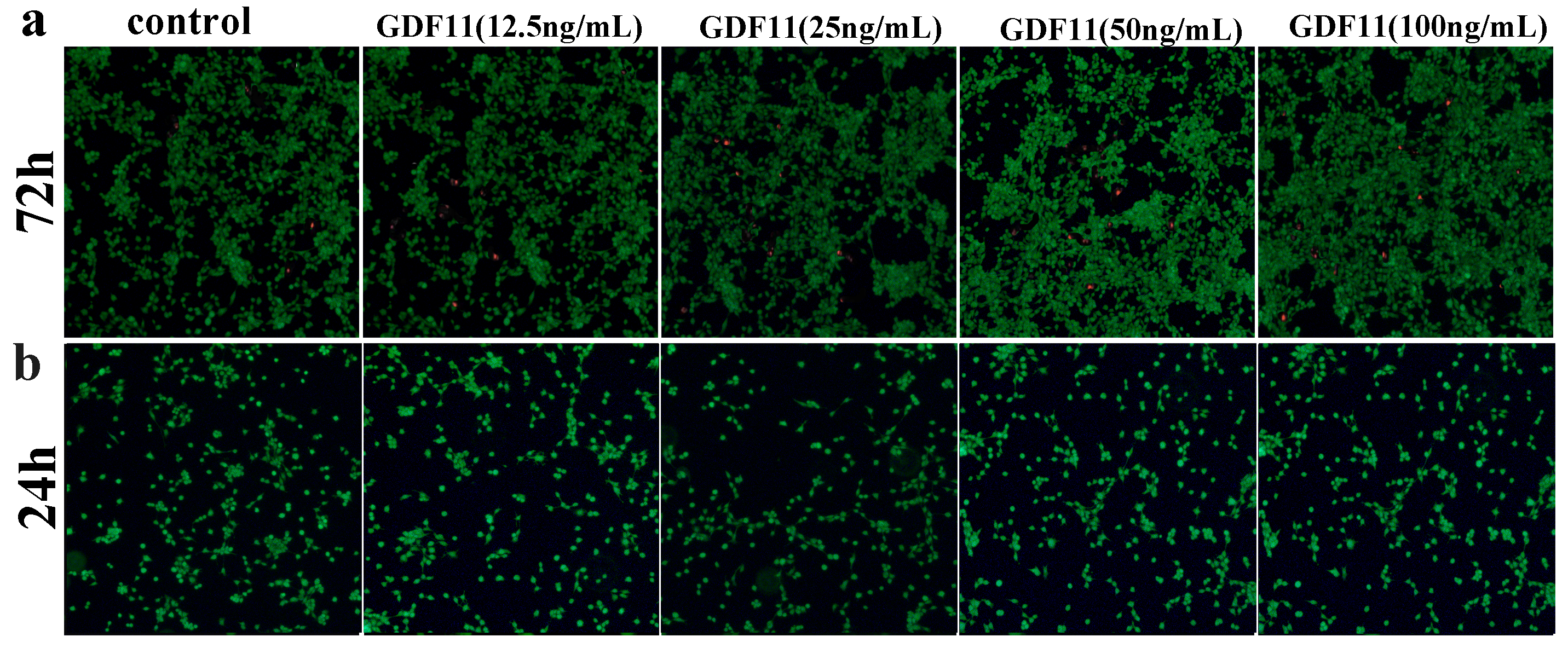

Supplement: Figure S1 — Images were obtainedat 50Xmagnification by inverted fluorescence microscope. The live cells were stained with calcein AM in green, and the dead cells were stained with EthD-1 in red. [file peerj-06-5524-s004.tif]

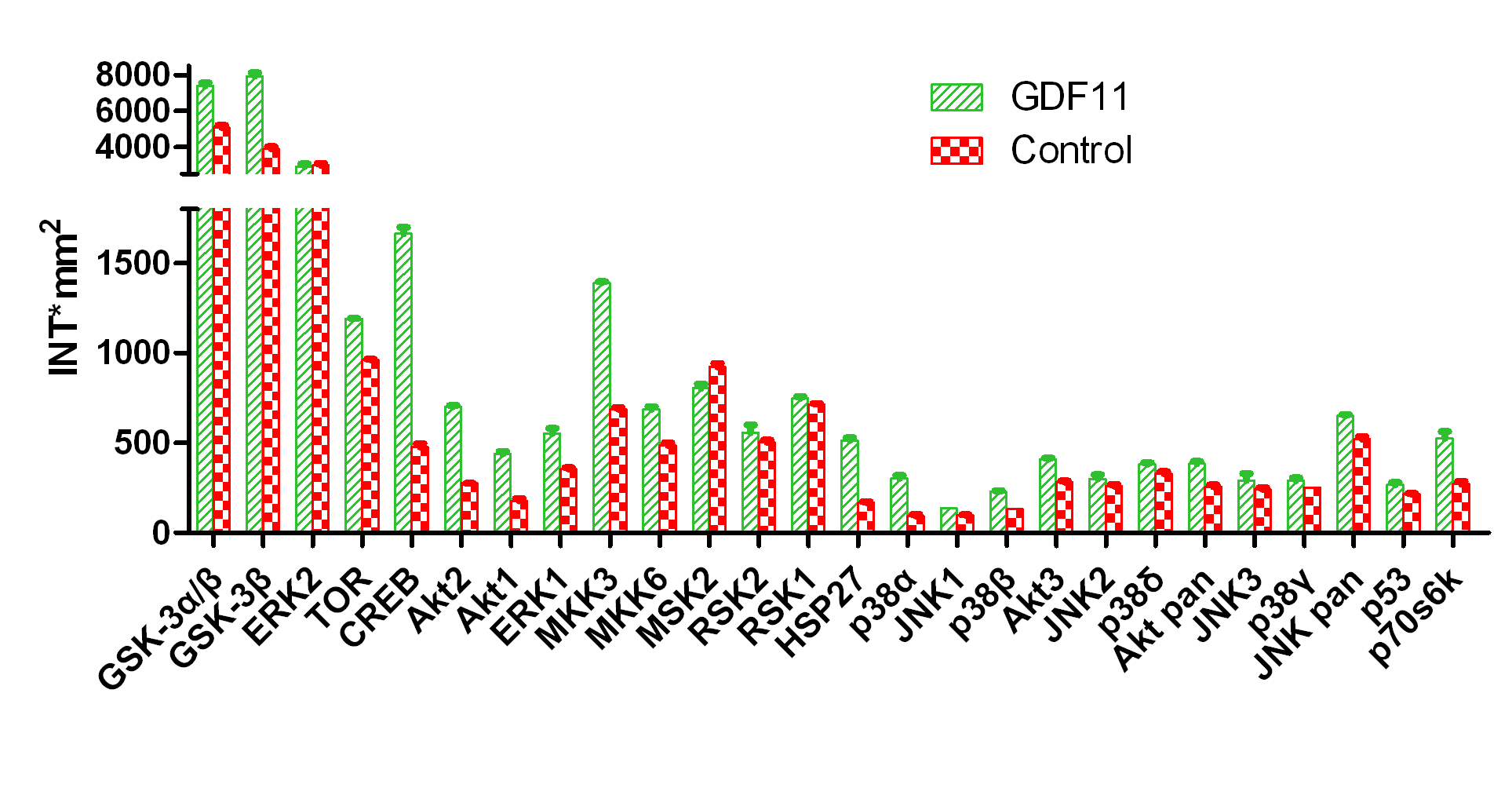

Supplement: Figure S2 [file peerj-06-5524-s005.tif]
