# Supplementary material for: GDF11 induces differentiation and apoptosis and inhibits migration of C17.2 neural stem cells via modulating MAPK signaling pathway
Source: PeerJ. 2018 Sep 4;6:e5524. doi: 10.7717/peerj.5524 (PMC6128255; doi:10.7717/peerj.5524)
Supplement: Data S2 [file peerj-06-5524-s007.pdf]

1. The uncropped blots of Fig.2b

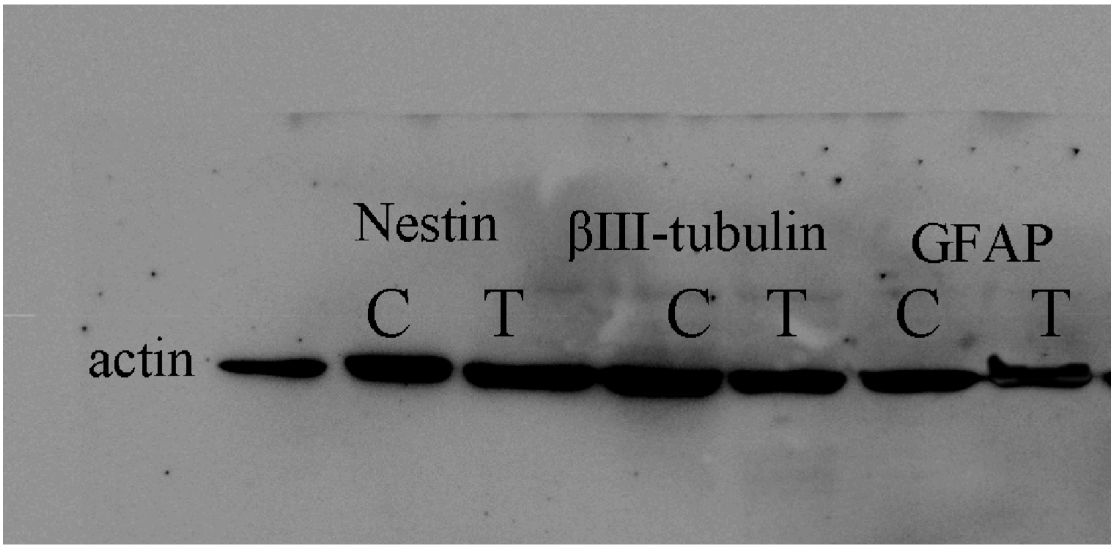

β-actin for Fig.2b

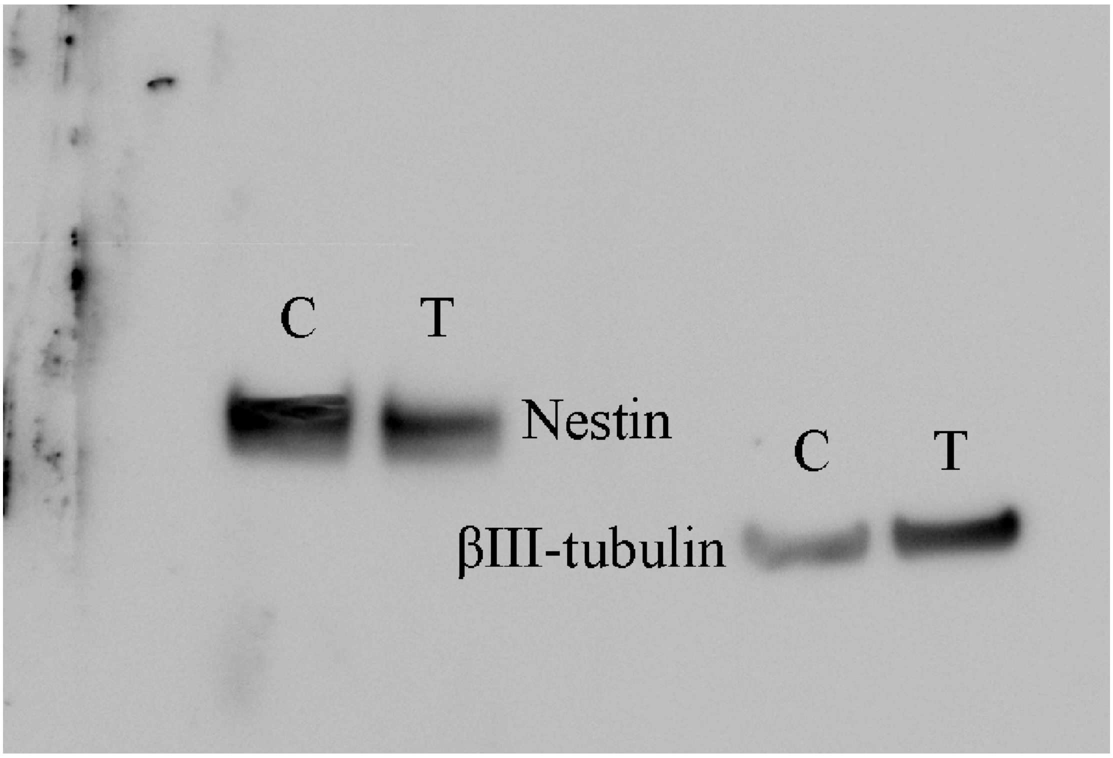

Nestin and β III-tubulin for Fig.2b

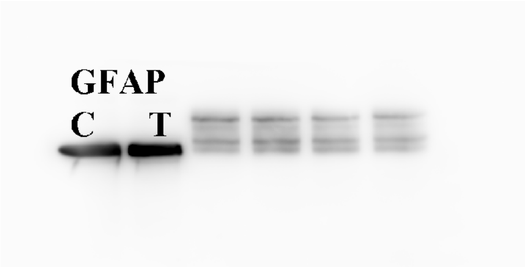

GFAP for Fig.2b

2. The uncropped blots of Fig.4

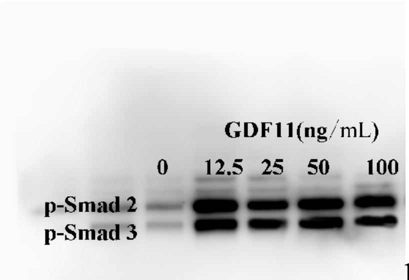

p-Smad2/3 for Fig.4a

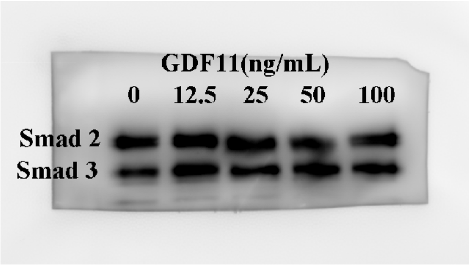

Smad2/3 for Fig.4a

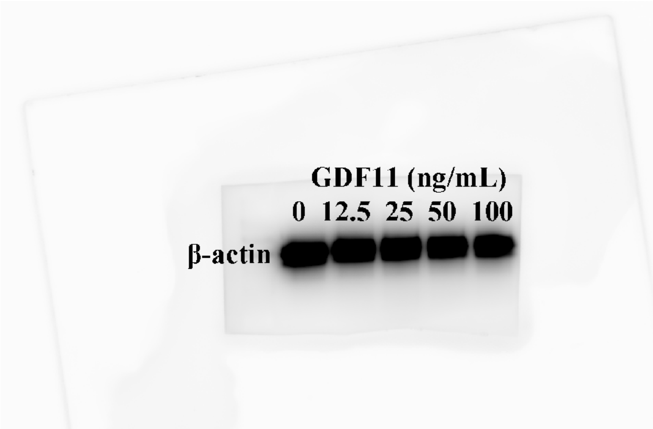

β-actin for Fig.4a

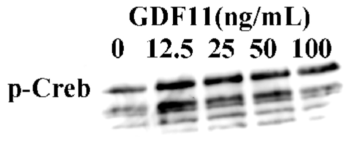

p-creb for Fig.4b

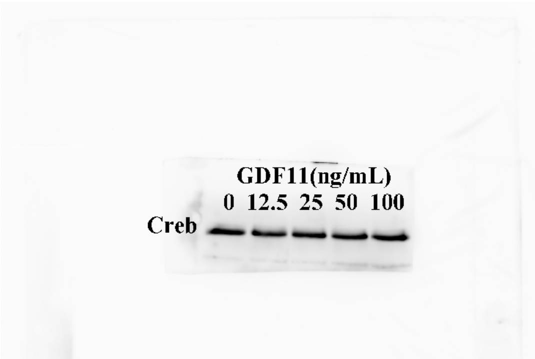

Creb for Fig.4b

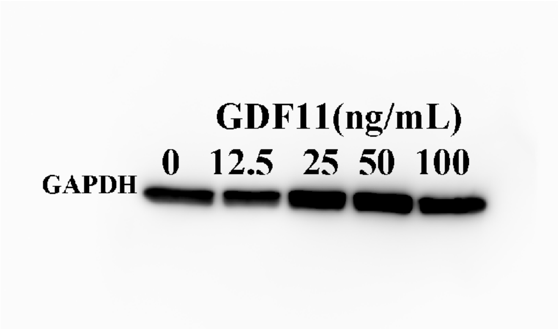

GAPDH for Fig.4b

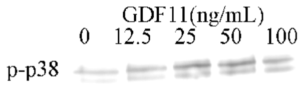

p-p38 for Fig.4c

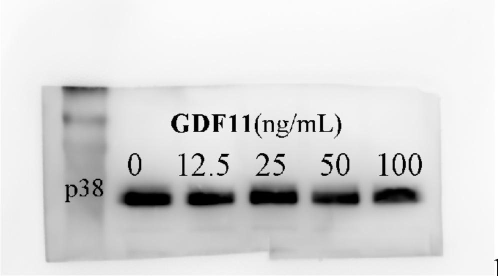

p38 for Fig.4c

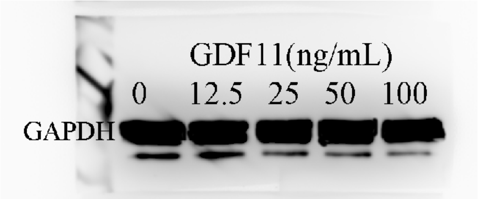

GAPDH for Fig.4c

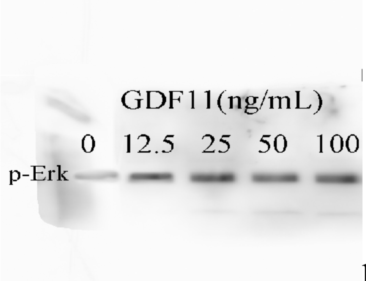

p-Erk1/2 for Fig.4d

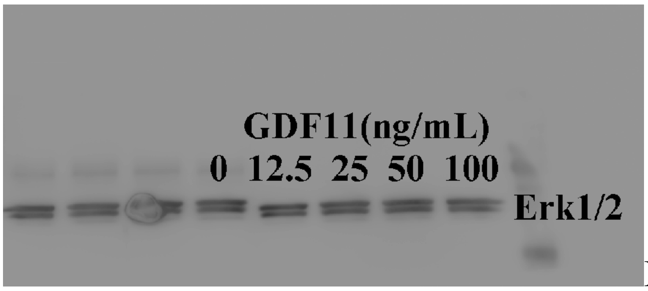

Erk1/2 for Fig.4d

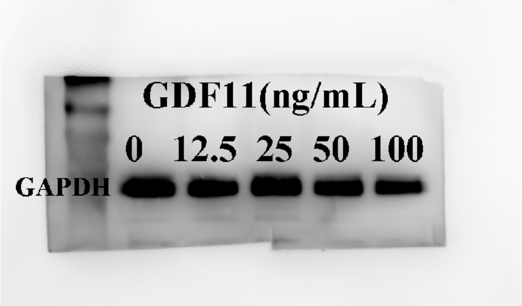

GAPDH for Fig.4d
